# Accumulation of Cotton in the Stomach

**Published:** 1850-05

**Authors:** 


ACCUMULATION OF COTTON IN THE STOMACH.—The following case may serve to show the extraordinary forbearance of the stomach, though far outdone by the celebrated clasp-knife case. We take it from that rich mine, the Boston Journal :

"Some three years since, a young woman, about twenty years of age, who worked in the cotton factory at Whitinsville, in this State, after having been for a long time pale, weak, and diseased in the stomach, was obliged to leave the factory and call for a physician. He found her without appetite, and greatly emaciated—great pain and suffering in the stomach.

On inquiry by her physician, Dr. Robbing, she stated that for several years, while laboring in the cotton mill, she had been accustomed to bite off threads, which it was necessary to remove, in the process of performing her task, and swallow them. Believing that an accumulation of this cotton in her stomach might be the cause of her suffering, Dr. R. gave her an emetic. By this and other means he succeeded in procuring from her stomach a quantity of cotton, which, when dried, weighed *four ounces*. Rev. Mr. Clark, of Whitinsville, who saw the cotton, and saw it weighed, observed, 'There was cotton enough to fill my hat half full.'"

*Ohio Med. and Surg. Journal.*

